# Fetal microchimerism, pregnancy epiphenomenon or kinship indicator?

**DOI:** 10.1098/rspb.2023.1906

**Published:** 2023-10-11

**Authors:** Graham J. Burton

**Affiliations:** Department of Physiology, Development and Neuroscience, University of Cambridge, Downing Street, Cambridge CB2 3EG, UK

When, in 1754, John Hunter demonstrated unequivocally that the maternal and fetal circulations are separated during pregnancy, the placenta became considered a barrier preventing the mixing of cells from the mother and her unborn child. However, since then it has been realized that the situation is more complex, and the bi-directional transfer of cells during pregnancy is now well established. Transfer of maternal cells to the fetus is referred to as maternal microchimerism, and that of fetal cells to the mother as fetal microchimerism. It is with the latter phenomenon that this Commentary, and the recent publication of Úbeda & Wild [1], is concerned.

The first demonstration of the presence of fetal cells within the mother is generally attributed to the pathologist George Schmorl. In 1893, he published his findings on the presence of small masses of multinucleated placental tissue occluding the pulmonary capillaries in women who died of eclampsia, a hypertensive complication of pregnancy. These thrombi are derived from the syncytiotrophoblast, the unique syncytial epithelium that covers the placental villous trees and interfaces with the maternal blood circulating through the placenta. Although they are part of a pathological process, it was recognized in the second half of the twentieth century that fetal leucocytes can be found circulating in the maternal blood during normal pregnancies from 7 weeks of pregnancy onwards. These cells are cleared rapidly after delivery. By contrast, fetal microchimerism refers to the maintenance of fetal cells with stem cell properties in the mother for several decades post-delivery. Such longevity allows fetal cells from successive pregnancies to accumulate within the mother, constituting her microchiome. Furthermore, fetal microchimeric cells from the first born can be passed on to subsequent siblings, and equally the mother may inherit microchimeric cells from her own mother, generating a grandmaternal effect [2]. This complexity challenges the concept of individuality, and the immunological premise of self and non-self. It also raises many questions as to the origin of the progenitor cells, their transplacental trafficking, their function, if any, and their immunological status.

The origin of the fetal cells is still uncertain and so they have been generically referred to as pregnancy-associated progenitor cells. They express the stem cell markers *POU5F1*/*OCT4*, *NANOG* and *REX1*, and those circulating in the maternal blood are CD34^+^ [3]. There are many potential sources present at the maternal–fetal interface; amniotic, trophoblastic, mesenchymal and endothelial stem cells have all been isolated from the placenta, while haematopoietic stem cells circulate through the placental capillaries in the fetal blood.

Fetal microchimerism has been reported in other species, and appears to be independent of the histological differences in the placental interface between mammals. In many species, the maternal uterine and fetal placental epithelia are simply in intimate apposition, with no invasion into the maternal tissues. This is referred to as epitheliochorial placentation [4]. At the opposite end of the spectrum is the arrangement seen in the human and great apes where the placenta invades into the maternal tissues, including her vascular system, so that maternal blood bathes the placental epithelium; haemochorial placentation. It might be assumed that the haemochorial situation favours fetal microchimerism as it allows for the most intimate access to the maternal circulation, but trafficking of fetal cells has been unequivocally demonstrated in the cow and pig, which have epitheliochorial placentas, and also in the dog that has an endotheliochorial placenta, intermediate in its invasiveness. A major factor limiting transplacental cell movement in the haemochorial and endotheliochorial situations is the presence of the syncytiotrophoblast. While in non-invasive placentas the trophoblastic epithelium covering the placenta is generally unicellular, in the invasive types the trophoblast cells fuse to form a multinucleated syncytial epithelium. In the human, this syncytium extends over the entire villous surface, approximately 12 m^2^ at term, and is a unique structure. The absence of intercellular clefts means that the syncytiotrophoblast represents a significant barrier to pathogens, such as *Listeria monocytogenes,* that are unable to penetrate [5]. The same is likely to be true for maternal and fetal cells. Thus, although it has been hypothesized that fetal cells may cross the placenta by a mechanism equivalent to that by which lymphocytes transmigrate across the blood-brain barrier [6], the evolutionary adaptation of a syncytial epithelium renders it extremely improbable. No images of cells transiting across an intact layer of syncytiotrophoblast have ever been published.

Defects in the integrity of the syncytiotrophoblast layer inevitably occur, however, due likely to physical interactions between villi and the impact of fetal movements. Areas of superficial damage are rapidly covered by a fibrin plug, but deeper areas may expose the villous core or even lead to fetal–maternal haemorrhage. Such injuries may release a mix of fetal cells, some with stem or progenitor properties, into the maternal circulation. Fetal cells have been detected in peripheral maternal blood in low numbers from as early as 7 weeks of pregnancy, increasing significantly at around 24 weeks and peaking at delivery [7]. This pattern suggests that most exchange occurs in late pregnancy, when the villous membrane separating the two circulations becomes more delicate, or at delivery when uterine contractions and separation of the placenta cause well-documented exchange between the circulations, and even mixing with amniotic fluid. Increased numbers of microchimeric cells are observed in pathological pregnancies, such as those complicated by fetal growth restriction, pre-eclampsia or pre-term labour. This finding may reflect aberrant placentation, or in the case of pre-eclampsia the greater degree of necrosis and shedding of the syncytiotrophoblast layer that occurs due to maternal malperfusion, weakening its barrier function.

The pregnancy-associated progenitor cells are thought to engraft into stem cell niches in the mother. Thus, male cells have been detected on the basis of *in situ* fluorescent hybridization for the Y chromosome, the easiest marker of microchimeric cells, in the bone marrow and also in the more accessible hair follicle, where they represented 0.4% of the stem cell population nearly two decades after a woman gave birth to her last son [8].

Differentiated cells bearing a Y chromosome have also been identified in all the major maternal organs, including the lungs, spleen, liver, kidney and heart in women who have given birth to a son or suffered miscarriage of a male fetus [9]. The highest incidence has consistently been found in the lungs, a distribution that may reflect the fact that this is the first capillary bed encountered by venous blood returning from the uterus. The cells appear morphologically identical to the mother's own cells, and to have been fully integrated physically and functionally into the parenchyma of the organ concerned.

When initially identified, the presence of the semi-allogeneic microchimeric cells was thought to explain the increased incidence of autoimmune disorders, such as systemic sclerosis and auto-immune thyroiditis, in post-partum women [10]. A contrary hypothesis soon emerged, that pregnancy-associated progenitor cells were being recruited along with the mother's own stem cells to areas of injury in an attempt at tissue repair [11]. The debate as to whether these cells are of benefit or danger to the mother, or both depending on maternal–fetal histocompatibility and the local microenvironment, continues to this day [12].

In their recent publication, Úbeda & Wild propose a new function for the microchimeric cells that will surely stimulate debate among reproductive and evolutionary biologists, their *information theory of microchimeric cells.* They hypothesize that the cells are involved in determining resource allocation to the fetus according to the mating behaviour of the mother [1]. The authors argue that by comparison of the genotype of the microchimeric cells with those within the existing maternal microchiome, either derived from earlier siblings or inherited from the grandmother, the cells inform the fetus of the likelihood that its genes will be present in future siblings. In cases of high likelihood resources are evenly allocated between the mother and her fetus, but in pregnancies where the maternal microchiome is genetically diverse it is argued that the fetus extracts a greater proportion of nutrients. The authors model their theory mathematically. How does this hypothesis fit with what is currently known about maternal–offspring resource allocation?

Appropriate resource allocation between the mother and her offspring is a key aspect of reproduction. The better nourished the offspring is the more likely it will survive and spread its genotype in the population. Fetal growth must be constrained in viviparous mammals, however, for the head must not grow so large that it cannot pass through the birth canal. Obstructed labour is a major cause of human maternal and perinatal mortality worldwide. The mother must also balance her interests in current and future offspring and not become too depleted during any one pregnancy. Multiple selection pressures are at work therefore. Currently, it is thought that parent–offspring resource allocation in both the animal and plant kingdoms is regulated primarily epigenetically through genes that are expressed on the basis of their parent of origin [13]. As a broad generalization, paternally expressed genes promote fetal growth, whereas maternally expressed genes act as a constraint. The balance between these two opposing forces acts as a rheostat that is capable of adapting to environmental conditions and balancing fetal nutrient demands to maternal supply.

Approximately 120 imprinted genes have been identified in the human where they are expressed in the placenta, the organ that interfaces between the mother and her fetus, and areas of the brain associated with reproduction. Imprinted genes can modulate nutrient transfer to the fetus through their effects on placental endocrine and transport functions. Placental hormones exert a powerful effect over maternal physiology during pregnancy; for example, secretion of growth hormone from the mother's pituitary gland is suppressed by mid-gestation by a variant produced from the placenta. Placental growth hormone-variant promotes breakdown of maternal fats and the synthesis of glucose, the principal energy substrate of the fetus and placenta, while antagonizing the peripheral effects of insulin. Other hormones influence maternal appetite and different aspects of her metabolism, all combining to increase the availability of nutrients for transfer to the fetus.

Nutrients cross the placenta by one of three principal routes; simple diffusion, facilitated diffusion and active transport. These pathways may be modulated during pregnancy to meet changes in environmental conditions or fetal demand; for example, the surface area for exchange or the density of transporter proteins inserted into the placental membrane may be increased or decreased. Genetic manipulation of fetal demand and placental supply in mouse models has revealed that fetally derived insulin-like growth factor 2, encoded by the imprinted gene *Igf2*, is a key demand signal and mediates its effects through its actions on protein synthesis and placental endocrine function [14].

In the hypothesis of Úbeda & Wild, it is not proposed that the microchimeric cells are directly involved in nutrient transfer, as has been suggested previously where they are considered as an extension of the endocrine placenta [15]. Instead, it is proposed that the cells act as an indicator of future kinship, and then feed this information back to the fetus. There are several aspects of the hypothesis that are challenging and will hopefully stimulate further research in the fields of evolutionary and reproductive biology. Firstly, the authors propose that the fetus is capable of sending variable numbers of cells across the maternal–fetal interface depending on the genetic diversity of the maternal microchiome. This implies an active process, but where the cells originate from and how they cross the interface are still uncertain. Although, as mentioned earlier, the current consensus is that accidental fetal–maternal haemorrhage is the most likely route, that is not to say there may be other, as yet unidentified, pathways that are more controllable.

Secondly, how and where is the genotype of the crossing microchimeric cells measured against that of the cells already present in the maternal microchiome? The authors suggest this may be through conventional receptor–ligand interactions. The most unique identifier of an individual is the expression of the highly polymorphic major histocompatibility antigens that form the basis of graft-host rejection. However, the longevity of the pregnancy-associated progenitor cells and of differentiated microchimeric cells in the mother suggest that they are able to evade immune surveillance by the maternal immune system or that the mother has become tolerogenic towards them in some way. How and when during the engraftment process this occurs is not known, and is an area ripe for further immunological research.

Thirdly, what is the resultant signalling intermediary and how is it relayed back to the fetus to instruct it to extract more or less nutrients from the mother? The authors suggest an autocrine factor, but the placenta has effective mechanisms for inactivating maternal hormones and inflammatory mediators so that the fetus develops in a neutral and stress-free milieu. The signal needs to bypass these defences in some way, and having done so influence nutrient extraction. It may operate through imprinted genes and the IGF2 axis, which has pleiotropic actions due to its effects on cell metabolism, but it does not necessarily have to. Placental hormones and most transporter proteins are not encoded by imprinted genes and so conceivably there could be more direct effects.

There is, therefore, still much to understand about both fetal and maternal microchimerism. It may be that the transfer of cells is an inevitable consequence of placentation, and that in the case of fetal microchimerism the migrating cells are in such a naive pluriopotent state that they are able to colonize the mother without undue reaction. Having entered the maternal stem cell pool they may then be stimulated to proliferate and mobilize along with her own stem cells to effect tissue repair. Alternatively, they may play a more active physiological role as hypothesized by Úbeda & Wild. Their hypothesis would seem to be eminently testable using outbreed mouse models with different mating diversities. If found to be valid the mechanistic steps could be elucidated, shedding important new light on maternal–fetal resource allocation. Whatever the outcome, the presence of a maternal microchiome and its heritability raises fascinating questions about individuality and kinship at many levels.

## Data Availability

This article has no additional data.
